# Phenolic Compounds of *Rumex roseus* L. Extracts and Their Effect as Antioxidant and Cytotoxic Activities

**DOI:** 10.1155/2021/2029507

**Published:** 2021-09-24

**Authors:** Mohamed Marouane Saoudi, Jalloul Bouajila, Khaled Alouani

**Affiliations:** ^1^Laboratoire de Chimie Analytique et Électrochimie, Faculté des Sciences de Tunis, Université de Tunis El Manar, Campus Universitaire 2092 Tunis, Tunisia; ^2^Laboratoire de Génie Chimique, Université Paul Sabatier, CNRS, INPT, UPS, Toulouse, France

## Abstract

*Rumex roseus* L. (*R. roseus*) is acknowledged as an aromatic plant. For its excellent biological properties, it was used as a traditional medicine. The aim of the present study is to evaluate the chemical components and their effect as the biological activities of Tunisian extracts of *R. roseus*. Consecutive extractions by cold maceration of the aerial part with solvents of increasing polarity (cyclohexane (CYH), dichloromethane (DCM), and methanol (MeOH)) were performed, and the different chemical groups (phenolics, flavonoids, tannins, anthocyanins, etc.) were identified. In addition, the volatile compounds of the obtained extracts were identified before and after derivatization. Moreover, their antioxidant and anticancer activities were evaluated. The analysis of HPLC-DAD revealed the identification of 18 components from organic extracts, among them are, for example, chlorogenic acid and shikonin, while GC-MS analysis allowed the detection of 34 volatile compounds. Some of those compounds were identified for the first time in plant extracts such as pyrazolo[3,4-d] pyrimidine-3,4(2H,5H)-dione (1); L-proline (16); 2-amino-3-hydroxybutanoic acid (19); L-(-)-arabitol (23); D-(-)-fructopyranose (25); and D-(+)-talopyranose (27). DPPH tests revealed that the most important antioxidant activity was found in the methanolic extract with 75.2% inhibition at 50 mg/L and that the highest cytotoxic activity against HCT-116 and MCF-7 was recorded in the dichloromethane extract with 62.1 and 80.0% inhibition at 50 mg/L, respectively. The biological activities were fully correlated with the chemical composition of the different extracts. So, we can suggest that *R. roseus* is a source of bioactive molecules that could be considered potential alternatives for use in dietary supplements for the prevention or treatment of diseases.

## 1. Introduction

As secondary plant metabolites, phenolic compounds are naturally found in all plant materials, to include plant-based food products [1]. Such compounds are generally thought to be an essential part of human and animal diets. However, they represent the largest known group of natural antioxidants [2]. Among the most common phenolic compounds contained in plants are phenolic acids, tocopherols, and flavonoids [3]. The antioxidant properties of these compounds are often claimed to be responsible for some forms of cancer and photosensitivity reactions; they have also the ability to inhibit the replication of human immunodeficiency virus (HIV), human simplex virus (HSV), glucosyltransferases of *Streptococcus mutans* (dental carries), and ascorbate autooxidation [4]. In addition, the most important characteristics of these compounds, notably flavonoids, include their ability to protect against oxidative diseases, to activate or inhibit various enzymes by binding to specific receptors, and to protect against cardiovascular diseases by reducing oxidation of low-density lipoproteins [5].

Building on past great successes in phototherapy, there are synergies between the use of medicinal plants, including fruits, vegetables, herbs, and spices, and the antioxidant power of bioactive molecules that constitute them [6, 7]. In recent years, natural substances are experiencing increasing interest in many areas. Indeed, with a general public increasingly reluctant to consume products containing molecules resulting from chemical synthesis, several industrial sectors (cosmetics, pharmaceuticals, and food processing) are once again turning to the inclusion of these molecules of natural source [8–10], which have original chemical and biological characteristics, in their formulations. The development of these active substances of natural origin therefore represents an enormous economic potential.

A significant diversity of species with multiple interests exists in Tunisia, including folk therapeutic practices, and many of them have not been chemically treated [11]. Tunisian flora is known for its diversity of medicinal plants such as *R. roseus* (Polygonaceae) commonly called “koressa.” Approximately 200 species of the genus *Rumex* are internationally distributed, and some of them are known for their phytoconstituents and traditional medicinal properties [12]. *R. roseus* grows spontaneously in Tunisia, and its leaves are consumed and enjoyed in cooked foods. It has anti-inflammatory, diuretic, astringent, purgative, and antispasmodic properties. In addition, this plant is used to reduce biliary disorders and control cholesterol levels [13].

Recent research shows that this plant contains a large number of phenolic compounds such as luteolin and ferulic acid which are very powerful reducing agents due to the presence of numerous OH- and C=O groups and also hydroxylated stilbenes, which are one of the most interesting and therapeutically important groups of plant polyphenols. Among them, trans-3,5,4′-trihydroxystilbene (trans-resveratrol) is detected in the stems of *Rumex roseus* [14].

Therefore, the presence of polyphenols in this plant, in particular chlorogenic acids, flavanols, flavones, phenolic acids, and tannins which are well known for their antioxidant properties [7], shows that it is suitable for use in certain fields such as the food industry. Furthermore, there are some reports in the literature on the evaluation of some other *Rumex* species that have shown health benefits and have been used as traditional foods and herbal remedies [15].

In order to search for new chemical compounds of *R. roseus* with potential antioxidant or antiproliferation activities, in this work, phytochemical screening (HPLC and GC-MS) of their extracts as well as their effect as antioxidant and cytotoxic activities were performed.

## 2. Materials and Methods

### 2.1. Chemicals and Reagents

All the chemicals, reagents, and standards employed in this study were of analytical quality (98.0-99.9%) and purchased from Sigma-Aldrich Chemical Company (Saint Quentin, France).

### 2.2. Plant Collection

The aerial part (stems, leaves, and flowers) of *R. roseus* was collected, from the locality of Borj-Cedria (Northern Tunisia). The plant was harvested in September 2014, corresponding to their full bloom period. Then, the harvested plant was identified at the center of biotechnology of Borj-Cedria (CBBC), and a voucher specimen (no. Rs014) was deposited at the herbarium of the Laboratory in the same center cited. To prepare the different extracts, 50 grams of fine powder was impregnated by using solvents of increasing polarity (500 mL for each solvent) for 2 hours. After filtering with the Whatman No. 2 filter paper (Fisher, France), the filtrate was evaporated on a vacuum rotary evaporator at 35°C (IKA, Germany). This program allows us to obtain dry extracts of CYH, DCM, and MeOH. Store these extracts at -4°C until further analysis (phytochemical and biological analysis). The extraction yield is characterized as follows: (*R*%) = (*m*_residue_/*m*)∗100, where *m*_residue_ is the weight of residue (g) and *m* is the weight of plant material (g).

### 2.3. HPLC-DAD Analysis

The different extracts of *R. roseus* were analyzed by analytical HPLC-DAD. This analysis was performed with the Dionex Ultimate 3000 pump and Thermos Separation Waters 996 product detector (Thermo Fisher Scientific, USA) using a reverse-phase C18 column (25 cm × 4.6 mm × 5 *μ*m) as described by Yahyaoui et al. [16], with some modifications.

The mobile phase used for elution is 1.2 mL/min with a limit of detection (LOD) defined between 0.01 and 0.1 mg/L. The mobile phase includes acidified water (pH = 2.65) (solvent A) and acidified water/ACN (20 : 80 *v*/*v*) (solvent B). The sample was eluted by the following linear gradient: from 0.1% B to 30% B for 35 minutes, from 30% B to 50% B for 5 minutes, from 50% B to 99.9% B for 5 minutes, and finally from 99.9% B to 0.1% B 15 minutes. Using the acidified water/ACN mixture (80 : 20 *v*/*v*), the different extracts were prepared at the same concentration (20 mg/mL) and then filtered through a Millex-HA 0.45 *μ*m syringe filter (Sigma-Aldrich). After injection of twenty microliters (20 *μ*L) of each sample and detection at 280 nm, phenolic compounds were identified by comparison with the retention time of some standards with known retention time.

### 2.4. GC-MS Analysis

Analysis by gas chromatography-mass spectrometry (GC-MS) was performed according to the procedure described by Rahmani et al. [17].

After solubilizing the extracts in their extraction solvents at 5 mg/mL, analysis of 2 *μ*L of each sample was performed using the Varian Saturn 2000 ion trap GC/MS system (Les Ulis, France) and the CP-3800 GC system equipped with a DB-5MS fused silica capillary column (5% phenylmethyl polyoxane, 30 × 0.25 mm, 0.25 *μ*m film thickness). Chromatographic conditions were 60-260°C, increased at a gradient of 5°C/min, and continued for 15 minutes under isothermal conditions of 260°C. A second gradient was used to reach 340°C at a rate of 40°C/min. The trap temperature was 250°C, and the transmission line temperature was 270°C. Perform quality scans from 40 to 650 *m*/*z*.

All molecules were identified by comparing both their retention index (RI) against C5-C24 *n*-alkanes obtained on a nonpolar DB-5MS column with those provided in the literature and their mass spectra with the NIST 08 database (National Institute of Standards and Technology).

#### 2.4.1. Derivatization Method

The method of derivatization has been described by Rahmani et al. [17], and some modifications have been made. In order to identify other compounds susceptible to be volatile, in a 2 mL vial, mix 150 *μ*L of 99% N,O-bis (trimethylsilyl)trifluoroacetamide (BSTFA)+1% chlorotrimethylsilane (TMCS) with 1 mL extract (5 mg/mL in the solvent tetrahydrofuran (THF)). Afterwards, the mixture was stirred for 30 seconds to increase solubility. After that, the reaction mixture was kept at 40°C for 15 minutes. Finally, ten microliters (10 *μ*L) of each derived solution were injected into the same GC-MS equipment and analyzed as described in the previous section to identify the volatile molecules of each extract.

### 2.5. Biological Activities

#### 2.5.1. DPPH Free Radical Scavenging Activity

The antioxidant activity was measured using the DPPH test. The quantitative estimation of radical scavenging ability was determined according to the methods described by Rahmani et al. [17]. Add 20 *μ*L of the diluted plant extract (50 *μ*g/mL) to 180 *μ*L of 0.2 mmol/L methanol DPPH solution in a 96-well microplate (MicroWell, Thermo Fisher Scientific, Asian, France). This mixture was incubated in the dark at room temperature for 25 min. Then, the absorbance of all samples was measured at 524 nm using a Thermo Fisher Scientific Multiskan GO spectrophotometer. The antioxidant activity of the extracts was expressed as percent inhibition (% inhibition) of the free radical scavenging activity of each sample using the following formula: %inhibition = [(*A*_blank_ − *A*_sample_)/*A*_blank_]∗100, where *A*_sample_ is the absorbance of the sample with DPPH^**•**^ solution and *A*_blank_ is the absorbance of the DPPH^**•**^ sample without the extract test. As a reference antioxidant, ascorbic acid was used.

#### 2.5.2. Cytotoxic Activity

Cytotoxic effects of different extracts on two different human cell lines, MCF-7 and HCT-116, were assessed in vitro according to the method described by Rahmani et al. [17]. The activity was assessed by a 3-(4,5-dimethylthiazol-2-yl)-2,5-diphenyltetrazolium bromide (MTT) colorimetric assay. The cells were plated in 96-well plates at a concentration of 3 × 10^4^ cells/well in 100 *μ*L; then, 100 *μ*L of the appropriate culture medium (DMEM, Sigma-Aldrich, USA) for MCF-7 or RPMI-1640 (Sigma-Aldrich, USA) for HCT-116 was added to each well that contains samples of various concentrations. The plate was then incubated at 37°C for 48 hours. Next, the supernatant was then removed, and the cells were treated with 50 *μ*L of MTT solution during incubation at 37°C for 40 minutes. Then, after eliminating the MTT solution, 50 *μ*L of dimethyl sulfoxide (DMSO) was added to dissolve the insoluble formazan crystals. The absorbance was measured at 605 nm. Tamoxifen was used as a positive reference. The cytotoxic effect of the extract was estimated based on the percentage of growth inhibition and calculated as follows: %inhibition = [(*A*_blank_ − *A*_sample_)/*A*_blank_]∗100.

### 2.6. Statistical Analysis

All measurements are made in quadruplicate. Use SPSS 20.1 (version IBM, 20.0.2004, USA, and http://Guru.com) to calculate the significance of the data by ANOVA (two-way analysis of variance). The statistical difference between the solvents is estimated by Tukey's test. All values *p* < 0.05 were considered statistically significant.

## 3. Results and Discussion

### 3.1. Extraction Yields

In this study, three different solvents, with different polarities, namely, CYH, DCM, and MeOH, were used for the extraction of the aerial part of *R. roseus*. The yields obtained of various extracts are shown in Table 1, while the MeOH was highlighted to have the highest yield with 10.16%, followed by the CYH extract with 1.1% and finally the DCM extract with 0.28%. In general, the yield of the polar extract (MeOH) was higher than those of the nonpolar extracts (CYH and DCM). These results were much better than that reported by Savran et al. [18] when they found 2.31 to 8.63% of yields obtained from successive extraction of acetone, methanol, and water of the *Rumex scutatus* aerial part, respectively; also, these results are more important than that determined by Abdel-Hameed et al. [19] obtained with methanolic maceration at room temperature with 9.6% as the extraction yield of the aerial part of *Rumex visicarius* L. The difference in the yield of extracts from different extracts may be related to the availability of extractable ingredients.

### 3.2. Phenolic Compounds of *R. roseus* Extracts

#### 3.2.1. HPLC-DAD Analysis

**Figure 1:**
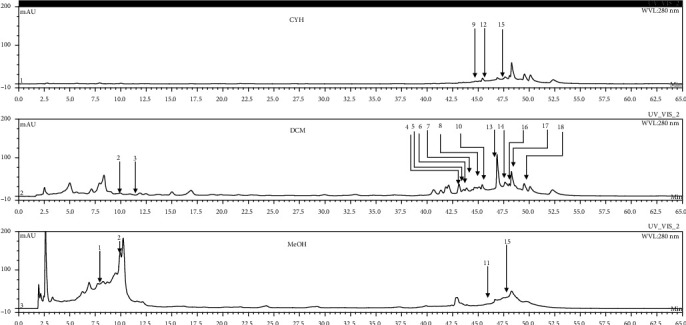
HPLC chromatogram profiles, visualized at 280 nm, of **CYH** (cyclohexane), **DCM** (dichloromethane), and **MeOH** (methanol) extracts obtained from aerial parts of *R. roseus* collected from Tunisia. Peaks: **(1)** 3,4-dihydroxy-5 methoxybenzoic acid; **(2)** chlorogenic acid; **(3)** (−)-epicatechin; **(4)** wedelolactone; **(5)** 5-hydroxy-7-((3-methylbenzyl) oxy)-2-phenyl-4h-chromen-4-one; **(6)** (z)-3-(3-ethoxy-4-hydroxy-phenyl)-2-phenyl-acrylic acid; **(7)** phenoxodiol; **(8)** pinostilbene hydrate; **(9)** ethyl trans-2-hydroxycinnamate; **(10)** caffeic acid 1,1-dimethylallyl ester; **(11)** 4-hydroxy-3-(3-oxo-1-phenylbutyl) coumarine; **(12)** cinnamyl-3,4-dihydroxy-*α*-cyanocinnamate; **(13)** pinosylvin monomethyl ether; **(14)** 3,6,3′-trimethoxyflavone; **(15)** shikonin; **(16)** 10-[(3-hydroxy-4-methoxybenzylidene)]-9(10H)-HMBA; **(17)** 5-hydroxyflavone; and **(18)** 3′-hydroxy-b-naphthoflavone.

**Figure 2:**
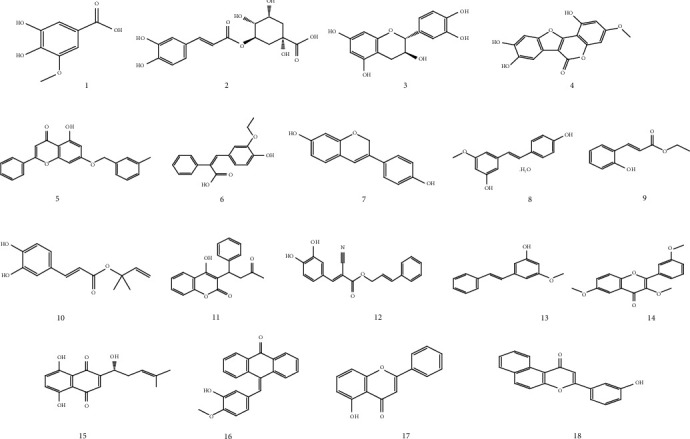
Chemical structures of identified metabolites listed in Table 2.

In order to identify the phenolic compounds extracted in the different extracts of *R. roseus*, 20 mg/mL of each extract was used by HPLC-DAD at 280 nm to visualize a maximum number of molecules at characteristic wavelength of phenolic compounds. The identification of each compound was based on the comparison of retention time with those found by standard compounds analyzed in the same condition. In total, 18 phenolic compounds were identified by means of their relative retention time as reported in Table 2. All these compounds were detected for the first time in extracts of *R. roseus.* When examining the chromatograms (Figure 1), it is observable that the chemical composition changes clearly qualitatively and quantitatively according to the polarities of solvent used. Indeed, the CYH extract contains more nonpolar phenolic compounds which their elution at the end of acquisition. As regards the DCM extract, both polar and nonpolar compounds can be recognized with maximum of intensity equal to 100. The chromatogram of the MeOH extract had more polar compounds eluted between 2 and 12 min compared to CYH and DCM extracts, and the maximum of intensity is 200. From this, we can deduce that the intensity of peaks increased with the polarity of solvent. To summarize, it is observable that the HPLC chromatograms showed that two compounds were detected in more than one extract, such as (2) chlorogenic acid and (15) shikonin (Figure 2). By comparison with the literature, most of the identified phenolic compounds were found for the first time in the *R. roseus* extracts. Some other phenolic compounds, such as (−)-epicatechin, were found in *Rumex acetosa* [20]. Moreover, Liu et al. [21] reported the existence of pinosylvin monomethyl ether and wedelolactone, respectively, in *Pinus pinaster* and *Ecliptae herba* extracts. These marked variations in chemical composition between the various organic extracts might be due to the influence of extractive power of each solvent. All these findings obtained by HPLC-DAD confirm that *R. roseus* extracts could be considered sources of valuable phytochemicals.

#### 3.2.2. Volatile Compounds by GC-MS Analysis

**Figure 3:**
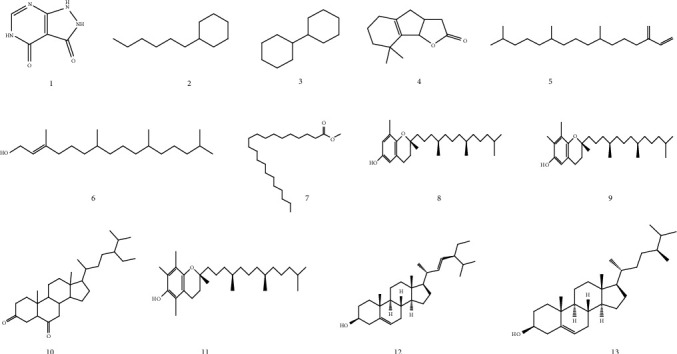
Structures of volatile compounds detected before derivatization, in the different extracts of *R. roseus*.

**Figure 4:**
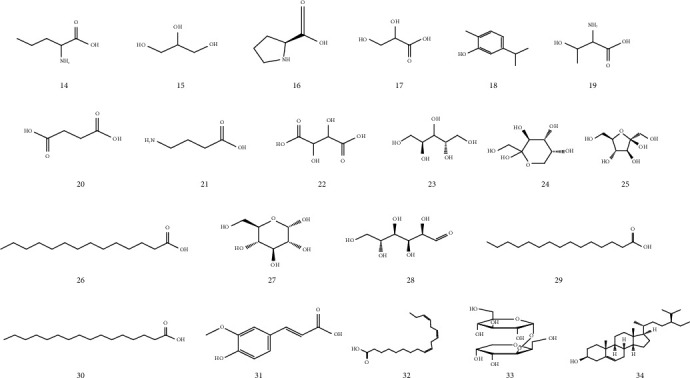
Structures of volatile compounds detected after derivatization, in the different extracts of *R. roseus*.

The compounds identified from GC-MS analysis of organic extracts from the aerial part of *R. roseus* are summarized in Table 3 and accompanied by their chemical structures (Figures 3 and 4). To our knowledge, this is the first study that analyzes the volatile compounds of organic extracts of *R. roseus*; thirteen volatile compounds were detected without derivation in the CYH and DCM extracts, while for the MeOH extract, only one volatile compound was identified. It is important to note that this research has enabled us to reveal that some compounds were detected in more than one extract, such as 7,11,15-trimethyl-3-methylidenehexadec-1-ene (5); methyl heneicosanoate (7); *α*-tocopherol (11); and *β*-sitosterol (13); this may be due to the fact that the compounds are released from the disturbed cells of the plant material by the maceration solvents under room temperature.

In order to identify more volatile compounds in the different extracts, a derivatization reaction was used to generate silylated products with chromatographic properties and volatility. However, this resulted in the identification of further 21 compounds in the three extracts, distributed as follows: two compounds in the CYH extract (26) and (29), one compound in the DCM extract, and linoleic acid (31) and 11 compounds identified only in the MeOH extract. However, the volatile profile of the different extracts indicates the presence of 7 classes of organic compounds: fatty acids, terpenes, phenols, sterols, vitamins, sugars, and aromatic hydrocarbons. According to an extensive literature review, it is important to emphasize that this research has revealed, for the first time, in *R. roseus* extracts the presence of molecules such as pyrazolo[3,4-d]pyrimidine-3,4(2H,5H)-dione (1); L-proline (16); 2-amino-3-hydroxybutanoic acid (19); L-(-)-arabitol (23); D-(-)-fructopyranose (25); and D-(+)-talopyranose (27).

Interestingly, MeOH contained a variety of phenolic compounds known for their antioxidant activity [22]. This result is consistent with the high antioxidant activity found in the current study that was developed thereafter. This type of GC-MS analysis is one of the steps towards understanding the nature of active compounds in medicinal plants. Because of the therapeutic properties of each of these compounds, this study should be pursued in order to develop methods for isolating these bioactive molecules that justify their use in food.

### 3.3. Antioxidant Activity

The antioxidant capacity of various extracts from the aerial part of *R. roseus*, harvested from Tunisia and prepared by cold maceration, was evaluated by the DPPH method. All the obtained results were expressed as inhibition percentage (%) and are displayed in Table 4. The best percentage has been recovered by the MeOH extract with 75.2%. However, the CYH and DCM extracts gave a weak activity. These results were compared to ascorbic acid (IC_50_ = 4.76 mg/L), used as a reference antioxidant. The high antioxidant effect observed in the MeOH extract could be attributed to the presence of some phenolic compounds which are known by their important biological effects especially in reducing DPPH free radicals. These results were higher compared to those found by Huyut et al. [5]; they found that the 3,4-dihydroxy-5 methoxybenzoic acid gives an important antioxidant activity with a percentage of inhibition equal to 98.64% at 20 *μ*g/mL. Also, the results of Parray et al. [23] revealed that shikonin showed 72% inhibition of radicals at 300 *μ*g/mL. On the other hand, some volatile compounds detected before derivatization in one or two extracts, like *β*-sitosterol, were shown to have antioxidant activity as well as interesting medicinal activity; they are able to improve cholesterol levels in the human body [24, 25]. We can note also that 5-isopropyl-2 methylphenol detected after derivatization is known for its significant antioxidant activities [26]. For this reason, phenolic compounds could be responsible for this activity. Compared to other *Rumex* species, previous studies have reported that the *Rumex crispus* extract showed more important antioxidant activity with lower inhibition concentrations with inhibition of 95.49% at a dose of 200 *μ*g/mL; the extract with hydromethanolic solvent of Indian *Rumex vesicarius* gives a 70% of inhibition, as well as the ethanolic Japanese *Rumex* (*Rumex japonicus*) (IC_50_ = 45 *μ*g/mL) [27–29].

Based on these findings, we argue that in order to evaluate a common activity such as DPPH radical scavenging, the use of different solvents could only lead to large result variability which varies according to the type of phenolic compounds extracted with each solvent.

### 3.4. Cytotoxic Activity

The results of the cytotoxic activity of the extracts are given in Table 4. All extracts were active against both cell lines, with the DCM extract being more dominant. By taking each cell line separately, a significant difference (*p* ≤ 0.05) was found between the different extracts, in terms of effect against each cell line. The MeOH extract had a weak effect against HCT-116 minus with an inhibition not exceeding 20%, whereas it showed good activity against the MCF-7 cell line. In addition, the CYH extract has an almost equal effect on both cell lines (HCT-116 and MCF-7) with inhibition of 46.1% and 54.4% at 50 mg/L, respectively. Moreover, for the two cell lines, HCT-116 and MCF-7, the DCM extract revealed the highest cytotoxic potential with, respectively, nearly 69.1 and 80.0% inhibition. This strong inhibition may be due to the presence of some phenolic compounds which are known for their important cytotoxic activities; some studies have suggested a potent activity of these compounds against cancer cells, especially against MCF-7 and HCT-116 cell lines, which is close to the obtained result. These results are consistent with the previously reported studies of its main active constituents including campesterol and *β*-sitosterol [30–32]. Also, Wang et al. [33] found that with the significant antioxidant activities exhibited by shikonin, it has shown also a powerful cytotoxic activity with IC_50_ equal to 0.83 and 0.53 (*μ*g/mL), respectively, for the two cell lines MCF-7 and HCT-116. Furthermore, these results of *R. roseus* extracts were higher than those found by other studies done on plants of the same family (*Polygonaceae*); they showed that the extract from the species *Rumex crispus*, for example, had no cytotoxic activity (0%) against the MCF-7 cell line [34] and in others as well (*Polygonum equisetiforme*); a low activity against both MCF-7 and HCT-116 was found, with 11.3 and 14.5%, respectively [35].

## 4. Conclusion

In this study, we have evaluated in vitro the antioxidant and anticancer activities of *R. roseus* areal part extracts. After studying the chemical compositions of all extracts, we can see that this plant contains significant amounts of phenolic compounds such as flavonoids, tannins, coumarins, and steroids that could provide scientific evidence for some popular uses in several fields. Based on the results obtained, we can conclude that the methanol extract could be considered an important natural antioxidant and that the DCM extract provides more effective cytotoxic activities for both cancer cells (HCT116 and MCF-7).

Further research needs to be conducted on the extracts of this plant in order to identify other molecules responsible for the biological activities.

## Figures and Tables

**Table 1:** Yields of the aerial part of *R. roseus* extracts.

Samples	Yields (%)
CYH extract	1.1
DCM extract	0.3
MeOH extract	10.2

CYH: cyclohexane; DCM: dichloromethane; MeOH: methanol.

**Table 2:** Phenolic compounds identified in various extracts of aerial parts of *R. roseus* by HPLC-DAD.

No.	Chemical compounds	RT (min)	Extracts			References
			CYH	DCM	MeOH	
1	3,4-Dihydroxy-5 methoxybenzoic acid	7.7	nd	nd	+	[5]
2	Chlorogenic acid	9.9	nd	+	+	[18]
3	(−)-Epicatechin	11.8	nd	+	nd	[16]
4	Wedelolactone	42.8	nd	+	nd	[21]
5	5-Hydroxy-7-((3-methylbenzyl) oxy)-2-phenyl-4h-chromen-4-one	43.0	nd	+	nd	[36]
6	(z)-3-(3-Ethoxy-4-hydroxy-phenyl)-2-phenyl-acrylic acid	43.0	nd	+	nd	[37]
7	Phenoxodiol	44.1	nd	+	nd	[38]
8	Pinostilbene	44.7	nd	+	nd	[39]
9	Ethyl trans-2-hydroxycinnamate	45.4	+	nd	nd	[40]
10	Caffeic acid 1,1-dimethylallyl ester	45.9	nd	+	nd	[41]
11	Warfarin	46.6	nd	nd	+	[42]
12	Cinnamyl-3,4-dihydroxy-*α*-cyanocinnamate	46.8	+	nd	nd	[43]
13	Pinosylvin monomethyl ether	47.1	nd	+	nd	[44]
14	3,6,3′-Trimethoxyflavone	47.8	nd	+	nd	[45]
15	Shikonin	48.0	+	nd	+	[46]
16	10-[(3-Hydroxy-4-methoxybenzylidene)]-9(10H)-HMBA	48.2	nd	+	nd	[16]
17	5-Hydroxyflavone	48.4	nd	+	nd	[47]
18	3′-Hydroxy-b-naphthoflavone	49.8	nd	+	nd	[16]

CYH: cyclohexane; DCM: dichloromethane; MeOH: methanol; nd: not detected; RT: retention time.

**Table 3:** GC-MS analyses before and after derivatization, in the different extracts of *R. roseus* aerial parts.

No.	Volatile compounds	RT (min)	CYH	DCM	MeOH
		Before derivatization			
1	Pyrazolo[3,4-d] pyrimidine-3,4(2H,5H)-dione	9.37	+	nd	nd
2	Cyclohexane hexyl	10.80	+	nd	nd
3	Bicyclohexyl	11.60	+	nd	nd
4	8,8-Dimethyl-3a,4,5,6,7,8b-hexahydro-3H-indeno[1,2-b] furan-2-one	13.43	nd	+	nd
5	7,11,15-Trimethyl-3-methylidenehexadec-1-ene	15.77	+	+	nd
6	Phytol	17.64	+	nd	nd
7	Methyl heneicosanoate	22.01	+	+	nd
8	*δ*-Tocopherol	25.24	+	nd	nd
9	*γ*-Tocopherol	26.02	+	nd	nd
10	Stigmastane-3,6-dione	26.76	nd	nd	+
11	*α*-Tocopherol	26.95	+	+	nd
12	Stigmasterol	28.52	+	nd	nd
13	*β*-Sitosterol	29.35	+	+	nd
		After derivatization			
14	2-Aminopentanoic acid	10.29	nd	nd	+
15	Glycerol	10.87	+	+	+
16	L-Proline	11.24	nd	nd	+
17	2,3-Dihydroxypropanoic acid	11.44	nd	nd	+
18	5-Isopropyl-2 methylphenol	11.62	+	+	nd
19	2-Amino-3-hydroxybutanoic acid	11.96	nd	nd	+
20	Butanedioic acid	12.92	+	++	+++
21	4-Aminobutanoic acid	13.31	nd	nd	+
22	2,3-Dihydroxysuccinic acid	14.06	+	+	nd
23	L-(-)-Arabitol	14.58	nd	nd	+
24	D-(-)-Fructopyranose	15.24	nd	nd	+
25	D-(-)-Fructofuranose	15.27	nd	nd	+
26	Myristic acid	15.83	+	nd	nd
27	D-(+)-Talopyranose	15.90	nd	nd	+
28	D-Glucose	16.45	nd	nd	+
29	Pentadecanoic acid	16.49	+	nd	nd
30	Palmitic acid	17.15	+	+	nd
31	Ferulic acid	17.50	nd	+	nd
32	Linoleic acid	18.19	+	+	nd
33	D-(+)-Turanose	20.38	nd	nd	+
34	Campesterol	28.06	+	+	nd

CYH: cyclohexane; DCM: dichloromethane; MeOH: methanol; nd: not detected; RT: retention time.

**Table 4:** Percentage inhibition (%), at 50 mg/L, of biological activities of *R. roseus* extracts.

Samples	Antioxidant activity	Cytotoxicity activity	
		HCT-116	MCF-7
CYH	16.87^b^ ± 1.1	46.1^a^ ± 10.7	54.4^b^ ± 1.2
DCM	18.5^b^ ± 2.3	62.1^a^ ± 9.0	80.0^a^ ± 1.6
MeOH	75.2^a^ ± 2.0	17.2^b^ ± 0.8	53.1^b^ ± 9.5
Vitamin C	95.3 ± 2.3	—	—
Tamoxifen (0.2 mg/L)	—	61.7 ± 4.2	47.17 ± 4.3

CYH: cyclohexane; DCM: dichloromethane; MeOH: methanol. Data are the mean of three repetitions ± SD. Different letters indicate significant differences according to the Tukey test (*p* ≤ 0.05).

## Data Availability

The datasets generated during the current PhD study are available from the corresponding author upon reasonable request.

## Abbreviations

ACN: Acetonitrile
CYH: Cyclohexane
DCM: Dichloromethane
DMEM: Dulbecco's modified Eagle's medium
DMSO: Dimethyl sulfoxide
DPPH: 1-1-Diphenyl-2-picryl hydrazyl
HCT116: Human colon cancer cells
HPLC: High-performance liquid chromatography
MCF-7: Human breast cancer cells
MeOH: Methanol
MTT: 3-[4,5-Dimethylthiazol-2yl]-2,5-diphenyl tetrazolium bromide
RPMI: Roswell Park Memorial Institute medium
SD: Standard deviation.
